# Oncolytic adenoviruses and the treatment of pancreatic cancer: a review of clinical trials

**DOI:** 10.1007/s00432-023-04735-w

**Published:** 2023-04-08

**Authors:** Isobel P. Taylor, J. Alejandro Lopez

**Affiliations:** 1 School of Medicine and Dentistry, Griffith University, Gold Coast, Australia; 2 School of Environment and Sciences, Griffith University, Nathan, Australia

**Keywords:** Adenovirus, Pancreatic cancer, Oncolytic virus

## Abstract

**Purpose:**

Pancreatic ductal adenocarcinoma (PDAC) remains a common and difficult cancer to treat. Surgical resection and chemotherapy are standard of care and clinical outcomes remain poor. Oncolytic adenoviruses are a unique approach to the treatment of this challenging cancer, aiming to overcome the features of this disease that pose the key obstacles to standard therapies. This paper provides a detailed review of the clinical trials of conditionally-replicative adenoviruses in pancreatic cancer to date, with a brief summary of the past preclinical literature and future prospects of this therapy.

**Methods:**

MEDLINE, Embase, and clinicaltrials.gov were searched from inception to December 23rd 2022 for clinical trials of conditionally-replicative adenoviruses used in patients with pancreatic ductal adenocarcinoma. Primary features for review included patient demographics, treatment protocol including dose and administration route, adverse events, patient responses and survival rates.

**Results:**

The six published clinical trials suggest that objective clinical responses can be achieved with a tolerable level of side effects, even at high viral doses. The more clinically adaptable intravenous route of administration also appears to be as well tolerated as the more challenging intratumoural injections.

**Conclusion:**

Published clinical trials provide data of the safety and some signs of oncolytic activity of conditionally-replicative adenoviruses in patients with pancreatic cancer. Importantly, on the latest trials, the easier intravenous route of administration seems to be well tolerated and safe, providing the opportunity for further clinical evaluation. It is hoped that the ongoing clinical trials will yield more promising results of this therapeutic approach against a currently intractable disease.

**Supplementary Information:**

The online version contains supplementary material available at 10.1007/s00432-023-04735-w.

## Introduction

Pancreatic ductal adenocarcinoma (PDAC) is a form of exocrine pancreatic cancer that accounts for more than 90% of all pancreatic cancer diagnoses and is the 12th most common cancer worldwide (Sung et al. 2021). The 5-year-survival rate is approximately 10% (Park et al. 2021). PDAC is usually diagnosed at advanced stage, with 30–35% of patients presenting with locally advanced disease and 50–55% of patients with metastatic disease (Park et al. 2021).

Pancreatic intraepithelial neoplasms acquire cumulative genetic insults which instigate oncogenes, such as KRAS, CDKN21, TP53 and SMAD4 that promote cancer development (Park et al. 2021). These mutated genes prevent innate tumour suppression and result in altered growth signalling and inflammation (Park et al. 2021). They also provide targets for oncolytic virotherapy, as they allow viruses to selectively replicate inside cells with such faulty pathways.

Surgical resection is the only curative alternative, although most patients present with unresectable disease. Current standard-of-care chemotherapy includes various combinations of gemcitabine, FOLFIRINOX, nab-paclitaxel, capecitabine and erlotinib (Principe et al. 2021) and has improved 5-year survival, with rates increasing over the past several decades, from 3.2% in 1987 to 10.7% in 2016 (Welfare 2022). This rate is significantly lower than the average 5-year survival rate achieved for all cancers, which was 69% in 2016 (Welfare 2022).

This high mortality rate is posited to be due to both the late stage at presentation, and the high resistance of PDAC to conventional chemotherapy. This resistance is, at least, partly due to the dense extracellular matrix that characterises PDAC. This environment distorts vessels, leading to hypoxic tumours that are inaccessible to chemotherapy which is typically delivered through the bloodstream. New approaches are needed to overcome the hostile PDAC microenvironment that may result in more effective treatments to almost 500,000 people diagnosed with pancreatic cancer every year (Sung et al. 2021).

Various forms of alternative/complementary treatments are being actively investigated. Radiation is being explored as an adjunct to chemotherapy option for locally advanced PDAC (Katz et al. 2017; Loehrer et al. 2011; Versteijne et al. 2020). Immunotherapies, including immune checkpoint inhibitors, immune agonists, cytokines and vaccines are also being explored as novel methods to overcome the immunosuppressive PDAC tumour microenvironment. These approaches have been described in recent reviews (Timmer et al. 2021; Zhu et al. 2022).

Oncolytic viruses offer an alternative approach to overcoming the challenges associated with treating PDAC. Clinical trials of oncolytic adenoviruses offer hope in improving outcomes for patients with this challenging cancer. Until recently, this approach was significantly limited by the very cumbersome and difficult intratumoral route of administration. Recent clinical trials using the intravenous route now facilitate the evaluation of this unique approach. This paper explores the use of oncolytic adenoviruses in clinical trials to date, with a brief summary of the preclinical trials.

### Oncolytic adenoviruses

Oncolytic viruses, or oncoviruses, preferentially kill tumour cells. They can be created through genetic modifications of existing viruses to ensure they selectively bind to and replicate in cancer cells. Several oncolytic viruses have been trialled in preclinical and clinical models of pancreatic cancer (Hamidi-Sofiani et al. 2022). Their biology has been utilised in two distinct ways.

*Replication-incompetent* viruses fail in one or more functions required for their replication, synthesis or the assembly of viral particles, and can be used as vaccines or vaccine vectors. They may be used to deliver cytokines or other cytotoxic molecules into the cancer cells. Alternatively, *replication-competent* viruses, capable of replicating, synthesising and assembling viral progeny can infect and reproduce effectively in cancer cells, leading them to elimination.

To optimise the use of oncoviruses for cancer therapy, two focal points are being improved upon in current research: selectivity and efficiency. The selectivity for cancer cells is improved by deleting the genes required to replicate in non-cancerous cells, by transcriptional or transductional targeting. Transcriptional targeting involves the use of tissue specific promoters on the virus to control replication. Transductional targeting is the retargeting of the virus specifically to tumour cells. These targeting strategies prevent oncoviruses from replicating in healthy cells, thereby making the virus selective for tumour cells. The efficiency of oncoviruses relies upon 3 factors; the ability to directly lyse cancer cells, the ability to migrate to surrounding cells and the ability to indirectly lyse cancer cells by promoting immune-mediated tumour cell death.

One family of viruses being investigated for use in PDAC are adenoviruses. These double-stranded DNA viruses are encompassed by an icosahedral protein capsid and can undergo mutations in order to improve their selectivity and efficiency as oncoviruses. Such recombinant adenoviruses enter cells when the fiber protein, which forms spikes on the corners of the capsid, binds to the coxsackie adenovirus receptor (CAR) with higher affinity than the receptor binds to itself, therefore hindering cell–cell adhesion and disrupting the tight junction. Inside the cell, the adenovirus genome is released and replicates until the cell disintegrates, hence lysing the cell and allowing progeny viruses to infect and lyse further cells.

Both deletional and transcriptional mutations have been used to create adenoviruses that are both selective for PDAC cells, and efficient at oncolysis, as have been previously reviewed (Nattress and Hallden 2018).

This review explores the modified adenoviruses that have been used in preclinical studies, as well as compiling and comparing reported clinical trials that have used oncolytic adenoviruses in PDAC.

## Summary of preclinical studies

Oncolytic adenoviruses have been studied in numerous preclinical models of pancreatic cancer. Of particular relevance are those modifications which have conferred increased tumour-selectivity and oncolytic efficiency, prompting their use in the design of clinical trials. Supplementary Table S1 shows a comprehensive list of the modifications explored in pre-clinical trials which have been reviewed further in recent papers (Nattress and Hallden 2018; Sato-Dahlman and Yamamoto 2018).

Of the various preclinical findings, some have been used in clinical trials. For example, deletions in the E1B region of the adenoviral genome improved tumour-selectively, as in ONYX-015 (Hecht et al. 2003; Mulvihill et al. 2001). The inclusion of the adenoviral death protein increases oncolytic activity and has been used in a clinical trial with Ad5-DS (Lee et al. 2020). Ad5-DS and a similar adenovirus, Ad5-yCD/mutTKsr39rep-hIL-12 have a yeast cytosine deaminase (yCD)/mutant sr39 herpes simplex virus thymidine kinase fusion gene which allows conversion of co-administered prodrugs to their active form within tumour cells, improving tumour cell lysis (Barton et al. 2021; Lee et al. 2020). In addition, the success of arming viruses with various cytokines has led their use in clinical trials. One example is Ad5-yCD/mutTKsr39rep-hIL-12 which harbours the immunostimulatory IL-12 (Barton et al. 2021).

E1A deletions have also been used in two clinical trials of VCN-01, as this deletion prevents viral replication in cells with functional pRb, therefore improving tumour-selectivity (Bazan-Peregrino et al. 2021; Garcia-Carbonero et al. 2022). VCN-01 also harbours RGD to allow binding and entry of the virus into cells with down-regulated CAR, such as tumour cells, therefore improving infectivity. Moreover, VCN-01 harbours E2F1 promoters in the E1A region to increase viral replication in tumour cells. Finally, human sperm hyaluronidase degrades the dense extracellular matrix of the tumour and was included in VCN-01 in recent clinical trials (Bazan-Peregrino et al. 2021; Garcia-Carbonero et al. 2022).

Ongoing clinical trials are also exploring the use of immunostimulatory modifications, such as CD40 ligand, 4-1BB ligand and interferon beta.

## Summary of clinical data

The great number of preclinical studies of adenovirus in models of pancreatic cancer have led to six clinical trials published to date, the majority of which in phase 1. Table 1 summarises the main features of each of these studies.

**Table 1:** Clinical trials exploring the use of oncolytic adenoviruses in PDAC

Title	Adenovirus	Other treatments	Route of administration	Phase	Dosage (viral particles)	Maximum tolerated dose (viral particles)	References
Safety and feasibility of injection with an E1B-55 kDa gene-deleted, replication-selective adenovirus (ONYX-015) into primary carcinomas of the pancreas: a phase I trial	**ONYX-015**—adenovirus with a 55 kDa deletion in the E1B region	N/A	Intratumoural (CT-guided + intraoperative)	I	6 doses ranging from 2 × 10^9^ to 2 × 10^12^	Not reached	Mulvihill et al. (2001)
A phase I/II trial of intratumoral endoscopic ultrasound injection of ONYX-015 with intravenous gemcitabine in unresectable pancreatic carcinoma	**ONYX-015**—adenovirus with a 55 kDa deletion in the E1B region	Gemcitabine	Intratumoural (endoscopic ultrasound-guided transduodenal and transgastric)	I–II	2 doses: 2 × 10^10^ and 2 × 10^11^	Not reached	Hecht et al. (2003)
Tolerability and safety of EUS-injected adenovirus-mediated double-suicide gene therapy with chemotherapy in locally advanced pancreatic cancer: a phase 1 trial	**Ad5-yCD/mutTKsr39rep-ADP**	Gemcitabine, 5-FC, valganciclovir, radiation	Intratumoural (endoscopic ultrasound-guided transgastric)	I	3 doses ranging from 10^11^ to 10^12^	Not reached	Lee et al. (2020)
Phase I trial of oncolytic adenovirus-mediated cytotoxic and interleukin-12 gene therapy for the treatment of metastatic pancreatic cancer	**Ad5-yCD/mutTKsr39rep-hIL-12**	5-FC, FOLFIRINOX or gemcitabine and albumin-bound paclitaxel	Intratumoural (endoscopic ultrasound-guided transgastric)	I	3 doses ranging from 10^11^ to 10^12^	Not reached	Barton et al. (2021)
VCN-01 disrupts pancreatic cancer stroma and exerts antitumor effects	**VCN-01**	Gemcitabine or nab-paclitaxel with gemcitabine	Intratumoural (endoscopic ultrasound-guided transgastric)	I	2 doses: 10^10^ and 10^11^	10^11^	Bazan-Peregrino et al. (2021)
Phase I, multicenter, open-label study of intravenous VCN-01 oncolytic adenovirus with or without nab-paclitaxel plus gemcitabine in patients with advanced solid tumors	**VCN-01**	Nab-paclitaxel and gemcitabine	Intravenous	I	2 doses: 3.3 × 10^12^ and 10^13^	10^13^	Garcia-Carbonero et al. (2022)

## Characteristics of the clinical trials

### Patient demographics

The cohort size in the studies ranged from 8 to 23 patients, and the median ages were similar, ranging from 60 to 68. All studies included both male and female patients with a male to female ratio ranging between 1:3 to 3:1. The functional status (expressed as the mean Eastern Cooperative Oncology Group (ECOG) score) varied from 0.33 to 0.67 between the studies. Arguably the most significant difference in patient demographics was in the tumour burden at the time of enrolment in the study. Barton et al. (2021) only included patients with distant metastases at baseline, whereas Lee et al. (2020) excluded all patients with distant metastases. The other 4 studies included patients with both locally advanced and metastatic disease (Table 2).

**Table 2:** Patient demographics

	Mulvihill et al. (2001)	Hecht et al. (2003)	Lee et al. (2020)	Barton et al. (2021)	Bazan-Peregrino et al. (2021)	Garcia-Carbonero et al. (2022)	
						Part 2	Part 3
Number of patients	23	21	9	12	8	12	14
Median age (range)	66.5 (33–79)	63 (34–78)	68 (49–71)	68 (55–84)	NR (50–70 +)	60 (35–75)	66 (49–86)
Female: male (%)	27:73	43:57	56:44	75:25	25:75	42:58	64:36
Mean ECOG^a^ score	Range 0.00–2.00^b^	0.50^b^	0.33	0.50	0.63	0.67	0.57
% with locally advanced disease	35	43	100	0	25	17	14
% with distant metastases	65	57	0	100	75	83	86
Mean tumour volume (range) (cm^3^)	18 (4–36)	NR	NR	22 (6–57)	NR	NR	NR
Median longest tumour diameter (cm)	NR^c^	NR	4.0	3.3	NR	NR	NR

^a^ECOG, Eastern Cooperative Oncology Group

^b^Converted from Karnofsky Performance Status Scores

^c^*NR* not reported

### Treatment

#### Previous treatments

Two studies (Lee et al. 2020; Barton et al. 2021) only included previously untreated patients, whereas all other studies included patients who had received previous chemotherapy, radiation, surgery or a combination of these therapies. The number of patients who had received no prior treatment ranged from 26 to 100% (Table 3).

**Table 3:** Previous treatments

	Mulvihill et al. (2001)	Hecht et al. (2003)	Lee et al. (2020)	Barton et al. (2021)	Bazan-Peregrino et al. (2021)	Garcia-Carbonero et al. (2022)^a^	
						Part 2	Part 3
Previous chemotherapy (%)	43.4	38.1	0.0	0.0	12.5	NR	NR
Previous radiation (%)	17.4	NR	0.0	0.0	0.0	NR	NR
Previous surgery (%)	52.2	NR	0.0	0.0	12.5	NR	NR
No previous treatment (%)	26.1	NR	100.0	100.0	75.0%	66.6	85.7

^a^In Garcia-Carbonero et al. (2022), 33.3% of patients in Part 2 and 14.3% of patients in Part 3 had previously received antineoplastic treatment, though the type is not reported

#### Adenovirus

The two earliest studies (Mulvihill et al. 2001; Hecht et al. 2003) investigated ONYX-015, which is an adenovirus with a deletion of the E1B-55 kDa region which allows it to replicate selectively inside cancer cells with abnormalities in the p53 pathway, including pancreatic ductal adenocarcinoma cells. This virus has been shown to be effective in preclinical models of PDAC (Bischoff et al. 1996; Heise et al. 1997).

The subsequent two studies (Lee et al. 2020; Barton et al. 2021) used similar adenoviruses. Lee et al. (2020) investigated Ad5-DS, which is an adenovirus with two suicide genes in the E1 domain: yeast cytosine deaminase and herpes simplex virus 1 thymidine kinase. These genes convert the prodrugs 5-FC and valganciclovir to 5-fluorouracil and valganciclovir-5-monophosphate respectively. These active metabolites lyse tumour cells. The Ad5 adenoviral death protein gene in the E3 region enhances the virus’s oncolytic capacity. Barton et al. (2021) uses Ad5-yCD/mutTKSR39rep-hIL-12, which expresses the two suicide genes as used in Lee et al. (2020), as well as human IL-12. The IL-12 acts to stimulate the immune system to improve oncolysis.

Finally, the most recent two studies (Bazan-Peregrino et al. 2021; Garcia-Carbonero et al. 2022) investigated VCN-01, another type 5 adenovirus that selectively replicates inside cells with retinoblastoma protein pathway deregulation, such as pancreatic ductal adenocarcinoma cells. The integrin-binding motif RGDK replaces the heparin sulfate glycosaminoglycan putative-binding site KKTK of the adenovirus 5 fiber, which increases tumour selectively and decreases liver tropism. Furthermore, VCN-01 expresses human sperm hyaluronidase that degrades the dense extracellular matrix that characterises pancreatic tumours to facilitate viral dissemination as well as chemotherapy infiltration.

#### Administration route

Mulvihill et al. (2001) used intratumoural administration, either under CT guidance or intra-operative injection. The subsequent 4 trials (Barton et al. 2021; Bazan-Peregrino et al. 2021; Hecht et al. 2003; Lee et al. 2020) used endoscopic ultrasound-guided intratumoural administration. The first trial to use this approach was Hecht et al. (2003). In this study both the trans-gastric and trans-duodenal approaches were utilised for virus administration, though two duodenal perforations occurred and subsequently the protocol was changed to mandate the trans-gastric approach. Finally, Garcia-Carbonero et al. (2022) was the first study to use systemic administration of an adenovirus for pancreatic cancer, via the intravenous route. Though there is a greater potential for systemic adverse events, this approach is less invasive and is more practical, especially if multiple administrations are to be used.

#### Dose

The first study of ONYX-015 in 2001 used doses between 2 × 10^9^ and 2 × 10^12^ viral particles and the maximum tolerated dose was not reached. The following study of ONYX-015 in 2003 used similar doses of 2 × 10^10^ and 2 × 10^11^ viral particles, with most patients (18 out of 21) receiving the higher dose. The maximum tolerated dose was also not reached.

In 2020, Ad5-DS was used at doses between 10^11^ and 10^12^ viral particles in Lee et al. (2020). The maximum tolerated dose was not reached. A similar adenovirus was studied in Barton et al. (2021) at the same doses as above and again the maximum tolerated dose was not reached.

The final adenovirus, VCN-01 was used at relatively lower doses, 10^10^ and 10^11^ viral particles in Bazan-Peregrino et al. (2021), and in this case the maximum tolerated dose was deemed to be 10^11^ viral particles. This suggests that VCN-01 causes more severe side effects at a lower dose than previously studied adenoviruses.

**Fig. 1:**
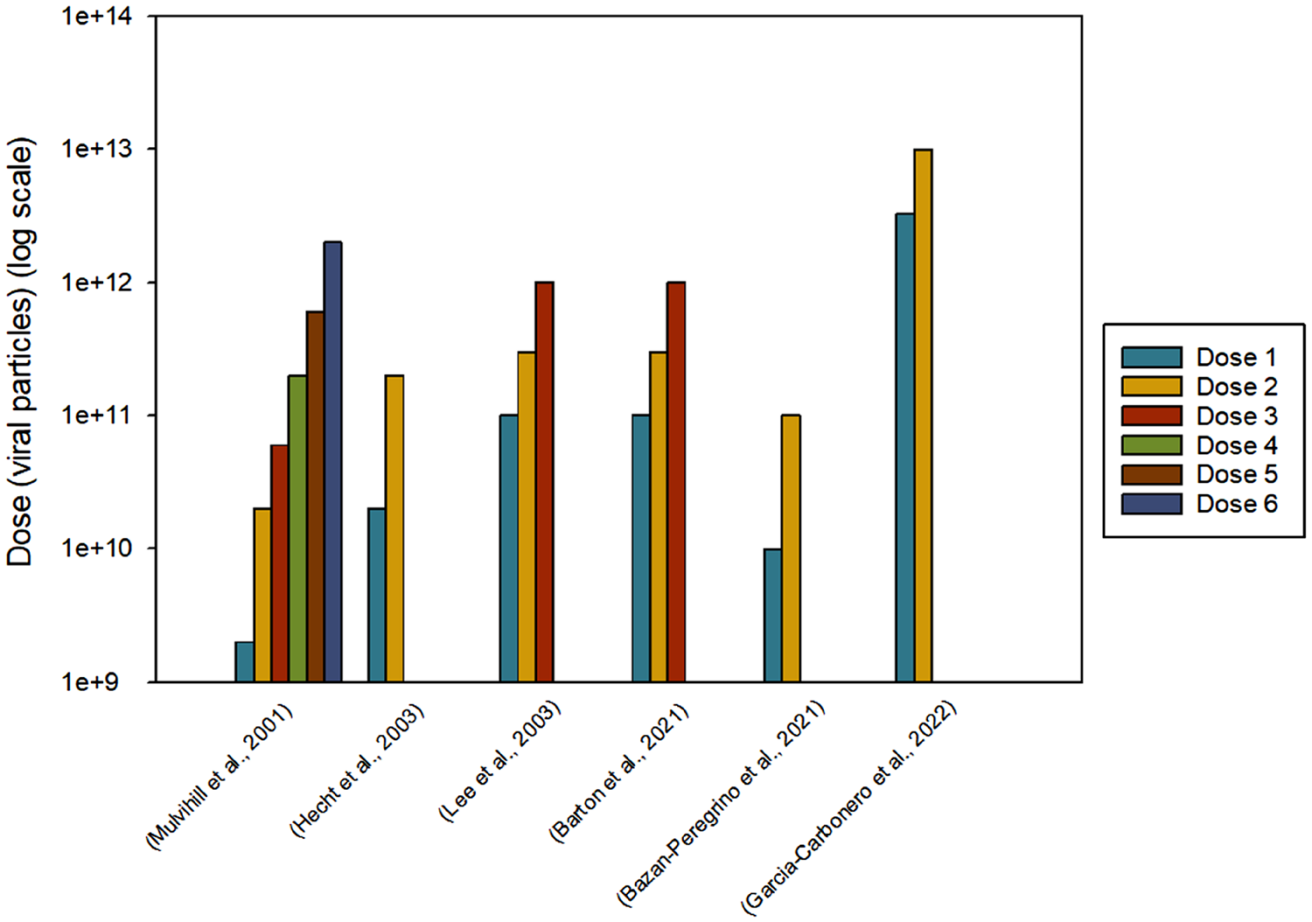
Doses of adenovirus used. Formatted using SigmaPlot version 15. The doses of adenovirus used in each clinical trial are shown on a logarithmic scale and in chronological order of the trial’s publication date. Most trials used two or three doses, whereas the first trial used six. Garcia-Carbonero et al. 2022 uses the largest doses of adenovirus of all of the trials and was the only trial to utilise the intravenous route

However, Garcia-Carbonero et al. (2022) studied VCN-01 at higher doses of 3.3 × 10^12^ and 10^13^ viral particles and the maximum tolerated dose was 100 times greater than in Bazan-Peregrino et al. (2021), at 10^13^ viral particles (Fig. 1).

#### Concomitant treatment

The protocol in Mulvihill et al. (2001) did not include concomitant treatments. The subsequent 5 papers included gemcitabine alone (Hecht et al. 2003; Bazan-Peregrino et al. 2021), gemcitabine, 5-FC and valganciclovir (Lee et al. 2020), 5-FC and FOLFIRINOX (Barton et al. 2021), 5-FC, gemcitabine and nab-paclitaxel (Barton et al. 2021) or gemcitabine with nab-paclitaxel (Bazan-Peregrino et al. 2021; Garcia-Carbonero et al. 2022). Lee et al. (2020) also reported that eight out of nine patients received stereotactic body radiation therapy during the study.

Barton et al. (2021) and Bazan-Peregrino et al. (2021) were the only studies that investigated the use of different concomitant chemotherapeutic drugs. Some patients in Barton et al. (2021) received FOLFIRINOX in place of gemcitabine and nab-paclitaxel, with both groups receiving 5-FC as well. In Bazan-Peregrino et al. (2021), some patients received nab-paclitaxel in addition to gemcitabine, whilst the others received gemcitabine as a single concomitant treatment.

Interestingly, Garcia-Carbonero et al. (2022) was the only study that investigated whether the timing of the concomitant chemotherapy in relation to the administration of the adenovirus would affect the success of the treatment or the adverse events. In this study, one arm (Part 2) received nab-paclitaxel and gemcitabine on the same day as the intravenous adenovirus infusion. In the other arm (Part 3), the adenovirus infusion occurred 1 week prior to the administration of the chemotherapy. This delay resulted in significantly fewer grade 3 or greater adverse events in Part 3 compared to Part 2.

#### Treatment schedule

Treatment schedules differed greatly between the six studies. In Mulvihill et al. (2001), ONYX-015 was administered every 4 weeks without concomitant chemotherapy. In Hecht et al. (2003), ONYX-015 was administered weekly as a single agent for 4 weeks and then in combination with gemcitabine for 4 weeks.

Patients in Lee et al. (2020) received treatment until disease progression or unacceptable toxicity. Each cycle consisted of a single Ad5-DS administration, then gemcitabine infusions two days, 9 days and 16 days later. Prodrugs 5-FC and valganciclovir were administered daily starting 2 days after adenovirus administration. The groups receiving the two lower viral doses had 7 days of prodrugs, whilst the group receiving the higher dose had 14 days.

In Barton et al. (2021), all patients received 7 days of 5-FC, beginning 48 h after adenovirus injection. In the 21 days following adenovirus injection, patients began receiving standard-of-care chemotherapy, such as FOLFIRINOX or gemcitabine with albumin-bound paclitaxel, at the discretion of the treating physician.

In Bazan-Peregrino et al. (2021), the lower adenoviral dose group received three injections of VCN-01 on days 1, 22 and 43 and gemcitabine on days 1, 8, 15, 22, 29, 35 and 43. Standard-of-care chemotherapy had changed to gemcitabine with nab-paclitaxel for the higher dose group, who received three injections of VCN-01 on days 1, 29 and 57, with gemcitabine and nab-paclitaxel on days 1, 8, 15 and then every 4 weeks. After day 56, patients continued to receive standard-of-care chemotherapy until disease progression, unacceptable toxicity, withdrawal of consent or at the discretion of the investigator.

Garcia-Carbonero et al. (2022) was the first study to explore the effects of a delayed administration of chemotherapy. In Part 2, the group received VCN-01 on the same day as chemotherapy, whereas the Part 3 group received the chemotherapy 7 days after the VCN-01 infusion. The schedule for Part 2 was a single injection of VCN-01 on day 1 with infusions of gemcitabine and nab-paclitaxel on the same day, followed by infusions of gemcitabine and nab-paclitaxel on days 8 and 15 of a 28-day cycle. Patients in Part 3 received VCN-01 on day 1, with infusions of gemcitabine and nab-paclitaxel on days 8, 15 and 22 of a 35-day cycle. Subsequent cycles for both groups were 28 days, with nab-paclitaxel and gemcitabine on days 1, 8 and 15.

### Adverse events

The oncolytic treatment was generally well tolerated with a low incidence of severe adverse events across the trials. It is difficult to analyse the trend in adverse events due to the different systems used to define adverse events and the differences in the reporting of adverse events in the six trials.

The system used to define treatment adverse events was not reported in the first two studies (Mulvihill et al. 2001; Hecht et al. 2003). Moreover, these two studies did not report the total number of adverse events but did explain that the majority of adverse events were not severe.

In the 4 subsequent papers, the Common Terminology Criteria for Adverse Events (CTCAE) grading was used.

In Lee et al. (2020), Bazan-Peregrino et al. (2021) and Garcia-Carbonero et al. (2022), the adverse events were reported according to the dose of adenovirus that the patients received. The other three papers did not classify the adverse events according to which dose level the patients had received (Table 4).

**Table 4:** Total adverse events (AEs) reported

	Mulvihill et al. (2001)	Hecht et al. (2003)	Lee et al. (2020)			Barton et al. (2021)	Bazan-Peregrino et al. (2021)		Garcia-Carbonero et al. (2022)	
									Part 2	Part 3
Dose (viral particles)	All	All	10^11^	3 × 10^11^	10^12^	All	10^10^	10^11^	All	All
Total AEs per patient^a^	NR^b^	NR	0.00	0.66	0.66	10.08	8.00	3.17	3.67	2.07
Total grade 1–2 AEs^a^	NR	NR	0.00	0.66	0.66	9.50	7.50	2.33	2.83	1.86
Total grade 3–4 AEs^a^	NR	1.00	0.00	0.00	0.00	0.58	0.50	0.67	0.75	0.21
Total grade 5 AEs^a^	NR	0.00	0.00	0.00	0.00	0.00	0.00	0.17	0.08	0.00
MTD^c^	Not reached	Not reached	Not reached	Not reached	Not reached	Not reached	Not reached	Not reached	10^13^	10^13^

^a^Calculated by dividing the total number of adverse events by the number of patients in the group

^b^NR, not reported

^c^MTD, maximum tolerated dose

In Lee et al. (2020), there were no adverse events reported in the patients who received the lowest viral dose. In the two higher doses, there were approximately 0.66 adverse events per patient on average, all of which were grade 1 or 2 in severity.

Barton et al. (2021) reported the greatest number of adverse events, with an average of approximately 10.08 events per patient. The vast majority of these were grade 1 or 2, with only 0.58 grade 3–4 adverse events per patient.

Bazan-Peregrino et al. (2021) interestingly reported a greater proportion of adverse events in the group who used the lower dose of the adenovirus compared to the higher dose (8.00 vs. 3.17 adverse events per patient). The proportion of grade 3 + severity events was greater in the group who received the higher dose of adenovirus (0.84 vs. 0.50 events per patient). Importantly, the group using the lower dose was comprised of only 2 patients, compared with the larger cohort of 6 patients in the higher dose group. Three dose-limiting toxicities occurred and so the maximum tolerated dose was deemed to be 10^11^, despite all other studies either not reaching the maximum tolerated dose or having a significantly higher maximum tolerated dose.

In Garcia-Carbonero et al. (2022), there were more adverse events reported in the group who received the adenovirus and the chemotherapy concurrently, compared to the group in which the adenovirus preceded the chemotherapy by 1 week (3.67 vs. 2.07 adverse events per patient). This trend was consistent across all grades of adverse events. Of particular importance, the group with the delayed chemotherapy regimen had significantly fewer reported grade 3 + adverse events (0.21 vs. 0.83 grade 3 + adverse events per patient).

The most commonly reported adverse events can be found in Table 5.

**Table 5:** Frequency of commonly reported adverse events

	Mulvihill et al. (2001)	Hecht et al. (2003)	Lee et al. (2020)			Barton et al. (2021)	Bazan-Peregrino et al. (2021)		Garcia-Carbonero et al. (2022)	
									Part 2	Part 3
Dose (viral particles)	All	All	1 × 10^11^	3 × 10^11^	1 × 10^12^	All	1 × 10^10^	1 × 10^11^	All	All
Fever/flu-like illness (%)	78	NR	0	0	66	50	50	50	67	86
Asthenia (%)	74	NR	NR	NR	NR	75	100	67	50	21
Thrombocytopaenia (%)	NR	NR	0	0	0	8	0	33	58	14
Anaemia (%)	22	NR	0	0	0	33	50	0	NR	NR
Leukopaenia (%)	21	NR	0	0	0	17	0	17	NR	NR
Nausea (%)	61	NR	0	0	0	67	0	33	25	21
Increased hepatic enzymes (%)	21	NR	NR	NR	NR	58	50	33	17	29

When the percentage of patients experiencing adverse events was not reported, the number of adverse events was divided by the number of patients to give an approximation

NR, not reported

Though these adverse events were common, very few patients experienced grade 3 + events.

It is important to note that Garcia-Carbonero et al. (2022) is the only study in which the adenovirus was administered systemically. Despite this, the proportion of total adverse events per patient, and the proportion of grade 3 + adverse events were less than those reported in the previous two studies, Bazan-Peregrino et al. (2021) and Barton et al. (2021).

### Clinical outcomes

The earlier studies in 2001 and 2003 investigated ONYX-015 and showed no objective responses observed when administered as a sole treatment. It was noted in Mulvihill et al. (2001) that whilst systemic dissemination of the virus occurred rapidly after administration, viral replication was not detectable thereafter. Perhaps this was due to the inflammatory and fibrotic PDAC microenvironment inhibiting viral spread, or due to hepatic or immunological clearance. In Hecht et al. (2003), gemcitabine was given alongside the final four administrations of adenovirus, and 9.5% of patients experienced partial regressions after this combination therapy, suggesting that dual chemotherapy-adenovirus treatments are more effective than adenovirus treatments alone. The response rate for gemcitabine alone, however, is 7% (Von Hoff et al. 2013), showing an inconsequential difference in response rates between chemotherapy alone and with adenovirus. All subsequent studies explored adenoviruses in combination with chemotherapy (Tables 6, 7, 8).

**Table 6:** Overall survival in the published trials, compared to standard treatments

	Dose (viral particles)	6-Month survival (%)	12-Month survival (%)	Median survival (range) months	References
Published trials	All	40	9	NR (range 2–18 +)	Mulvihill et al. (2001)
	All	67	29	7.5 (NR—36 +)	Hecht et al. (2003)
	All	100	NR	NR	Lee et al. (2020)
	10^11^	0	0	3.5 (2.7–10.5)	Barton et al. (2021)
	3 × 10^11^	33	0	4.8 (1.6–5.4)	
	10^12^	83	83	18.1 (3.5–20.0)	
	All	25	12.5	NR (0.7–31 +)	Bazan-Peregrino et al. (2021)
	All (Part 2)	NR	NR	11.0 (5.0–48.4)	Garcia-Carbonero et al. (2022)
	All (Part 3)	NR	NR	13.5 (2.6–29.6 +)	

	First-line agent	6-Month survival (%)	12-Month survival (%)	Median survival (95% CI) months	References
Standard therapies	FOLFIRINOX	75.9	48.4	11.1 (9.0–13.1)	Conroy et al. (2011)
	Gemcitabine + Nab-paclitaxel	67	35	8.5 (7.9–9.5)	Von Hoff et al. (2013)
	Gemcitabine	55	22	6.7 (6.0–7.2)	Von Hoff et al. (2013)

**Table 7:** Patient responses in the clinical trials that used RECIST V.1.1 criteria

	Lee et al. (2020)			Bazan-Peregrino et al. (2021)	Garcia-Carbonero et al. (2022)	
					Part 2	Part 3
Dose (viral particles)	10^11^	3 × 10^11^	10^12^	All	All	All
Total follow-up duration	6.5 months			31 + months	48 + months	29.6 + months
Timing of CT evaluations	12 weeks and 6.5 months			Every 8 weeks	Every 8 weeks	
Complete response (%)	0.0	0.0	0.0	0.0	8.3	0.0
Partial response (%)	0.0	33.3	0.0	12.5	33.3	42.9
Stable disease (%)	100.0	33.3	66.6	75.0	41.7	42.9
Progressive disease (%)	0.0	33.3	33.3	0.0	0.0	0.0
Not evaluable (%)	0.0	0.0	0.0	12.5	16.7	14.3

**Table 8:** Comparison of the patient responses in the clinical trials that used criteria other than RECIST v.1.1

	Mulvihill et al. (2001)						Hecht et al. (2003)	Barton et al. (2021)		
Dose (viral particles)	10^8^	10^9^	3 × 10^9^	10^10^	3 × 10^10^	10^11^	All	10^11^	3 × 10^11^	10^12^
Follow-up duration	Median 5.5 months						Median 6 weeks	Median 16.0 months		
Timing of CT evaluations	Every 22 days						Day 35, 63 and then every 6–8 weeks	Every 2 months		
Minor response/partial response (%)	33.3	33.3	66.6	16.7	0.0	0.0	9.5	0.0	0.0	0.0
Stable disease (%)	50.0	33.3	0.0	50.0	66.6	100.0	38.1	0.0	66.7	50.0
Progressive disease (%)	16.7	33.3	33.3	33.3	0.0	0.0	52.4^a^	66.7	33.3	50.0
Not evaluable (%)	0.0	0.0	0.0	0.0	33.3	0.0	0.0	33.3	0.0	0.0

*NR* not reported

^a^This figure includes patients who had to leave the study due to toxicity

The more standardised evaluation of clinical outcomes (RECIST) has been applied to four of the trials and they yielded some encouraging data. A different adenovirus (Ad5-DS) was explored in Lee et al. (2020) and 11.1% of patients experienced a partial response, which where the most promising results yet. This study did, however, exclude patients with metastatic disease and therefore the participants had better prognoses than participants in the other studies. Adenoviral DNA was detected in serum at 8 weeks in 44.4% of patients, suggesting viral replication had occurred.

In contrast, Barton et al. (2021) investigated a similar adenovirus to Lee et al. (2020), but exclusively recruited patients with distant metastases. The poor prognosis of the patients included likely contributed to the poor survival and response rates seen, with no objective responses reported.

Bazan-Peregrino et al. (2021) and Garcia-Carbonero et al. (2022) both explored another different adenovirus, VCN-01. When administered via the intratumoural route in Bazan-Peregrino et al. (2021), 12.5% of patients had a minor response and the mean time to progression was 8.4 months. In contrast, Garcia-Carbonero et al. (2022) was the first study to investigate the less invasive intravenous route as well as the effect of administering the adenovirus on the same or different day as the chemotherapy. In the group that received VCN-01 and chemotherapy on the same day, a higher objective response rate (10% compared to 0%) and a longer median time to progression (9.9 months compared to 6.7 months) were observed. The objective response rates of both groups in Garcia-Carbonero et al. (2022) were less than that of Bazan-Peregrino et al. (2021), and so perhaps the intravenous route of administration is less effective than the intratumoural route. Despite this, a second peak in plasma viral concentration as well as increased serum hyaluronidase was seen in Garcia-Carbonero et al. (2022), suggesting viral replication occurred in all patients.

While more difficult to compare, there are encouraging data arising from trials where criteria other than RECIST were used for the evaluation of clinical outcomes. In Mulvihill et al. (2001), a minor response is defined as a 25–49% decrease in the cross-sectional area of the tumour. Stable disease is defined as an increase or decrease of less than 25% in the cross-sectional area of the tumour. Progressive disease is defined as an increase of equal to or greater than 25% in the cross-sectional area of the tumour.

Hecht et al. (2003) reports that responses were evaluated using standard WHO criteria. Using these criteria, a partial response involves a decrease of greater than or equal to 50% in the sum of products of the two longest diameters in perpendicular dimensions (Park et al. 2003). This is a significantly greater response than is defined as a minor response in Mulvihill et al. (2001).

## Discussion

Preclinical studies of oncolytic adenoviruses have shown promise of these agents in models of pancreatic cancer. Clinical trials offer encouraging results, including the favourable safety profiles of the four adenoviruses used. The most common adverse events included fever, nausea and asthenia, which are events commonly associated with the current standard treatment for pancreatic cancer, gemcitabine. Moreover, the majority of the reported adverse events were grade 1–2, suggesting the severity of such events was mild and patients tolerated the adenoviruses well. Of significance, the intravenous route utilised in Garcia-Carbonero et al. (2022) appears to be safe and well-tolerated. This trial did not report the highest number of adverse events, as might be expected with systemic administration of the adenovirus. Additionally, the higher dose used in the trial was up to 5000 times greater than doses used previously, yet the number and severity of adverse events was in keeping with the previous trials. The patient responses were also encouraging with the majority of patients achieving at least stable disease throughout the surveillance period. Survival rates varied significantly even between patients receiving the same viral dose and treatment schedule, perhaps owing to the small sample sizes of the trials, individual variation in disease severity or other unreported factors. Larger studies are needed to explore the effectiveness of this therapy in more detail now that the safety and tolerability of intratumoural and intravenous adenovirus administrations have been established.

Despite the promising results shown in the published trials, there are challenges of the human PDAC microenvironment that pose barriers to treatment with oncoviruses and have limited the effectiveness of this treatment. Firstly, viral selectivity is reduced when there is widespread distribution of primary cellular receptors as systemic toxicity limits the viral dose that may be tolerated.

The route of administration poses another challenge, as the commonly used intratumoural administration is relatively invasive and less suitable to target distant metastases than the intravenous route. This method itself poses the theoretical significant risk of liver tropism, which could cause considerable adverse effects and reduce the availability of adenovirus in the circulation. However, Garcia-Carbonero et al. (2022) has now shown that an oncolytic adenovirus may be administered via the intravenous route at doses significantly greater than previously used, whilst remaining tolerable with comparable adverse effects to the intratumoural delivery.

The significant host immune response triggered by adenoviruses can enhance oncolysis in the local tumour environment but can also reduce efficiency and safety when a systemic reaction occurs. Modifying the adenovirus such that is largely or exclusively delivered into tumour cells is necessary to prevent such adverse reactions.

Furthermore, the dense fibrotic microenvironment that characterises PDAC prevents traditional chemotherapeutic agents from penetrating the tumour, and similarly prevents efficient adenovirus replication and spread. This issue is difficult to overcome, but Bazan-Peregrino et al. (2021) and Garcia-Carbonero et al. (2022) have attempted to overcome this through expression of hyaluronidase on the oncolytic adenovirus to degrade the extracellular matrix and facilitate the spread of viral progeny and chemotherapies throughout the tumour. A phase III trial of pegvorhyaluronidase, however, showed no improvement in overall survival or progression free survival in patients with metastatic pancreatic cancer, posing doubts about the utility of viral hyaluronidase expression (Van Cutsem et al. 2020).

Whilst there are many challenges to overcome, conditionally-replicative adenoviruses offer an innovative therapy for a cancer that is difficult to treat.

Further advances in the construction and administration of adenoviruses aim to build upon the promising results seen in the clinical trials. For example, the adenovirus library approach, which involves the display of random peptides on a fiber knob, allows generation of adenovirus that can target specific cell types, allowing researchers to synthesise adenoviruses with affinities for specific cancers (Yamamoto et al. 2014).

Arming adenoviruses with cytokines has also been shown to increase the oncolytic ability of oncoviruses, without compromising safety.

Ongoing clinical trials are summarised in supplementary table 2. These trials include adenoviruses which have been trialled before, such as Ad5-yCD/mutTKSR39rep-ADP which was studied in Lee et al. (2020), and adenoviruses which have not yet been explored in clinical trials of pancreatic cancer.

Many other strategies are also being explored to improve the efficacy of oncolytic adenoviruses without modifying the viral genome. For example, administering the virus within mesenchymal stromal carrier cells, monocytes and lymphocytes encourages viral production by reducing the immune response to the virus (Bunuales et al. 2012; Hammer et al. 2015; Kaczorowski et al. 2016; Na et al. 2019; Santos et al. 2021). Creating a complex of oncolytic adenovirus and cationic nanoparticles also enhances infection and spread (Man et al. 2022). Oncolytic adenoviruses can be chemically conjugated with neurotensin-conjugated PEF to reduce immunogenicity and improve tumour selectivity through the neurotensin receptor (Na et al. 2015). Hydrogel-based delivery of oncolytic adenoviruses is being explored as an additional method to overcome the immune reaction to systemic administration of adenovirus (Du et al. 2022).

Finally, alternative therapies, such as domperidone, have been shown to enhance the replicative ability of oncolytic adenoviruses in PDAC (Nishimae et al. 2022). At the preclinical stage, there are a myriad of avenues by which the oncolytic efficacy of adenoviruses is being improved in order for these viruses to offer a practical and effective option in the treatment of pancreatic cancer. The developments already tested in the clinical trials reported here have yielded very encouraging results whereby significant clinical objective responses have been achieved in a disease which progression is normally linear. A combination of novel viral modifications and the evaluation of safer and effective routes of administration to be tested in the ongoing clinical trials is likely to further enhance the potential of this therapeutic approach.

## Supplementary Information

Below is the link to the electronic supplementary material.

Supplementary file 1 (PDF 184 KB)

## Data Availability

The datasets generated during and/or analysed during the current study are available from the corresponding author on reasonable request.

## References

[CR1] Barton KN, Siddiqui F, Pompa R, Freytag SO, Khan G, Dobrosotskaya I, Ajlouni M, Zhang Y, Cheng J, Movsas B, Kwon D (2021) Phase I trial of oncolytic adenovirus-mediated cytotoxic and interleukin-12 gene therapy for the treatment of metastatic pancreatic cancer. Mol Ther Oncolytics 20:94–104. 10.1016/j.omto.2020.11.006 33575474 10.1016/j.omto.2020.11.006 PMC7851493

[CR2] Bazan-Peregrino M, Garcia-Carbonero R, Laquente B, Alvarez R, Mato-Berciano A, Gimenez-Alejandre M, Morgado S, Rodriguez-Garcia A, Maliandi MV, Riesco MC, Moreno R, Ginesta MM, Perez-Carreras M, Gornals JB, Prados S, Perea S, Capella G, Alemany R, Salazar R, Hidalgo M (2021) VCN-01 disrupts pancreatic cancer stroma and exerts antitumor effects. J Immunother Cancer. 10.1136/jitc-2021-003254 10.1136/jitc-2021-003254 PMC8578996 35149591

[CR3] Bischoff JR, Kirn DH, Williams A, Heise C, Horn S, Muna M, Ng L, Nye JA, Sampson-Johannes A, Fattaey A, McCormick F (1996) An adenovirus mutant that replicates selectively in p53-deficient human tumor cells. Science 274(5286):373–376. 10.1126/science.274.5286.373 8832876 10.1126/science.274.5286.373

[CR4] Bunuales M, Garcia-Aragoncillo E, Casado R, Quetglas JI, Hervas-Stubbs S, Bortolanza S, Benavides-Vallve C, Ortiz-de-Solorzano C, Prieto J, Hernandez-Alcoceba R (2012) Evaluation of monocytes as carriers for armed oncolytic adenoviruses in murine and Syrian hamster models of cancer. Hum Gene Ther 23(12):1258–1268. 10.1089/hum.2012.043 22985305 10.1089/hum.2012.043 PMC3523252

[CR5] Conroy T, Desseigne F, Ychou M, Bouche O, Guimbaud R, Becouarn Y, Adenis A, Raoul JL, Gourgou-Bourgade S, de la Fouchardiere C, Bennouna J, Bachet JB, Khemissa-Akouz F, Pere-Verge D, Delbaldo C, Assenat E, Chauffert B, Michel P, Montoto-Grillot C, Intergroup P (2011) FOLFIRINOX versus gemcitabine for metastatic pancreatic cancer. N Engl J Med 364(19):1817–1825. 10.1056/NEJMoa1011923 10.1056/NEJMoa1011923 21561347

[CR6] Du YN, Wei Q, Zhao LJ, Fan CQ, Guo LR, Ye JF, Li Y (2022) Hydrogel-based co-delivery of CIK cells and oncolytic adenovirus armed with IL12 and IL15 for cancer immunotherapy. Biomed Pharmacother 151:113110. 10.1016/j.biopha.2022.113110 10.1016/j.biopha.2022.113110 35605298

[CR7] Garcia-Carbonero R, Bazan-Peregrino M, Gil-Martin M, Alvarez R, Macarulla T, Riesco-Martinez MC, Verdaguer H, Guillen-Ponce C, Farrera-Sal M, Moreno R, Mato-Berciano A, Maliandi MV, Torres-Manjon S, Costa M, Del Pozo N, Martinez de Villarreal J, Real FX, Vidal N, Capella G, Salazar R (2022) Phase I, multicenter, open-label study of intravenous VCN-01 oncolytic adenovirus with or without nab-paclitaxel plus gemcitabine in patients with advanced solid tumors. J Immunother Cancer. 10.1136/jitc-2021-003255 10.1136/jitc-2021-003255 PMC8961117 35338084

[CR8] Hamidi-Sofiani V, Rakhshi R, Moradi N, Zeynali P, Nakhaie M, Behboudi E (2022) Oncolytic viruses and pancreatic cancer. Cancer Treat Res Commun 31:100563. 10.1016/j.ctarc.2022.100563 10.1016/j.ctarc.2022.100563 35460973

[CR9] Hammer K, Kazcorowski A, Liu L, Behr M, Schemmer P, Herr I, Nettelbeck DM (2015) Engineered adenoviruses combine enhanced oncolysis with improved virus production by mesenchymal stromal carrier cells. Int J Cancer 137(4):978–990. 10.1002/ijc.29442 25604186 10.1002/ijc.29442

[CR10] Hecht JR, Bedford R, Abbruzzese JL, Lahoti S, Reid TR, Soetikno RM, Kirn DH, Freeman SM (2003) A phase I/II trial of intratumoral endoscopic ultrasound injection of ONYX-015 with intravenous gemcitabine in unresectable pancreatic carcinoma. Clin Cancer Res 9(2):555–561. https://www.ncbi.nlm.nih.gov/pubmed/12576418 12576418

[CR11] Heise C, Sampson-Johannes A, Williams A, McCormick F, Von Hoff DD, Kirn DH (1997) ONYX-015, an E1B gene-attenuated adenovirus, causes tumor-specific cytolysis and antitumoral efficacy that can be augmented by standard chemotherapeutic agents. Nat Med 3(6):639–645. 10.1038/nm0697-639 9176490 10.1038/nm0697-639

[CR12] Kaczorowski A, Hammer K, Liu L, Villhauer S, Nwaeburu C, Fan P, Zhao Z, Gladkich J, Gross W, Nettelbeck DM, Herr I (2016) Delivery of improved oncolytic adenoviruses by mesenchymal stromal cells for elimination of tumorigenic pancreatic cancer cells. Oncotarget 7(8):9046–9059. 10.18632/oncotarget.7031 10.18632/oncotarget.7031 PMC4891025 26824985

[CR13] Katz MHG, Ou FS, Herman JM, Ahmad SA, Wolpin B, Marsh R, Behr S, Shi Q, Chuong M, Schwartz LH, Frankel W, Collisson E, Koay EJ, Hubbard JM, Leenstra JL, Meyerhardt J, O'Reilly E, Alliance for Clinical Trials on O (2017) Alliance for clinical trials in oncology (ALLIANCE) trial A021501: preoperative extended chemotherapy vs. chemotherapy plus hypofractionated radiation therapy for borderline resectable adenocarcinoma of the head of the pancreas. BMC Cancer 17(1):505. 10.1186/s12885-017-3441-z 10.1186/s12885-017-3441-z PMC5530569 28750659

[CR14] Lee JC, Shin DW, Park H, Kim J, Youn Y, Kim JH, Kim J, Hwang JH (2020) Tolerability and safety of EUS-injected adenovirus-mediated double-suicide gene therapy with chemotherapy in locally advanced pancreatic cancer: a phase 1 trial. Gastrointest Endosc 92(5):1044–1052 e1041. 10.1016/j.gie.2020.02.012 10.1016/j.gie.2020.02.012 32084409

[CR15] Loehrer PJ Sr, Feng Y, Cardenes H, Wagner L, Brell JM, Cella D, Flynn P, Ramanathan RK, Crane CH, Alberts SR, Benson AB 3rd (2011) Gemcitabine alone versus gemcitabine plus radiotherapy in patients with locally advanced pancreatic cancer: an Eastern Cooperative Oncology Group trial. J Clin Oncol 29(31):4105–4112. 10.1200/JCO.2011.34.8904 21969502 10.1200/JCO.2011.34.8904 PMC3525836

[CR16] Man YKS, Aguirre-Hernandez C, Fernandez A, Martin-Duque P, Gonzalez-Pastor R, Hallden G (2022) Complexing the oncolytic adenoviruses Ad and Ad-3-A20T with cationic nanoparticles enhances viral infection and spread in prostate and pancreatic cancer models. Int J Mol Sci. 10.3390/ijms23168884 10.3390/ijms23168884 PMC9408166 36012152

[CR17] Mulvihill S, Warren R, Venook A, Adler A, Randlev B, Heise C, Kirn D (2001) Safety and feasibility of injection with an E1B-55 kDa gene-deleted, replication-selective adenovirus (ONYX-015) into primary carcinomas of the pancreas: a phase I trial. Gene Ther 8(4):308–315. 10.1038/sj.gt.3301398 11313805 10.1038/sj.gt.3301398

[CR18] Na Y, Choi JW, Kasala D, Hong J, Oh E, Li Y, Jung SJ, Kim SW, Yun CO (2015) Potent antitumor effect of neurotensin receptor-targeted oncolytic adenovirus co-expressing decorin and Wnt antagonist in an orthotopic pancreatic tumor model. J Control Release 220(Pt B):766–782. 10.1016/j.jconrel.2015.10.015 26471393 10.1016/j.jconrel.2015.10.015

[CR19] Na Y, Nam JP, Hong J, Oh E, Shin HC, Kim HS, Kim SW, Yun CO (2019) Systemic administration of human mesenchymal stromal cells infected with polymer-coated oncolytic adenovirus induces efficient pancreatic tumor homing and infiltration. J Control Rel 305:75–88. 10.1016/j.jconrel.2019.04.040 10.1016/j.jconrel.2019.04.040 PMC6659406 31071373

[CR20] Nattress CB, Hallden G (2018) Advances in oncolytic adenovirus therapy for pancreatic cancer. Cancer Lett 434:56–69. 10.1016/j.canlet.2018.07.006 29981812 10.1016/j.canlet.2018.07.006

[CR21] Nishimae F, Sakurai F, Ono R, Onishi R, Takayama K, Mizuguchi H (2022) A dopamine antagonist, domperidone enhances the replication of an oncolytic adenovirus in human tumour cells. J Gen Virol. 10.1099/jgv.0.001752 10.1099/jgv.0.001752 35731650

[CR22] Park JO, Lee SI, Song SY, Kim K, Kim WS, Jung CW, Park YS, Im YH, Kang WK, Lee MH, Lee KS, Park K (2003) Measuring response in solid tumors: comparison of RECIST and WHO response criteria. Jpn J Clin Oncol 33(10):533–537. 10.1093/jjco/hyg093 14623923 10.1093/jjco/hyg093

[CR23] Park W, Chawla A, O’Reilly EM (2021) Pancreatic cancer: a review. JAMA 326(9):851–862. 10.1001/jama.2021.13027 34547082 10.1001/jama.2021.13027 PMC9363152

[CR24] Principe DR, Underwood PW, Korc M, Trevino JG, Munshi HG, Rana A (2021) The current treatment paradigm for pancreatic ductal adenocarcinoma and barriers to therapeutic efficacy. Front Oncol 11:688377. 10.3389/fonc.2021.688377 10.3389/fonc.2021.688377 PMC8319847 34336673

[CR25] Santos J, Heinio C, Quixabeira D, Zafar S, Clubb J, Pakola S, Cervera-Carrascon V, Havunen R, Kanerva A, Hemminki A (2021) Systemic delivery of oncolytic adenovirus to tumors using tumor-infiltrating lymphocytes as carriers. Cells. 10.3390/cells10050978 10.3390/cells10050978 PMC8143525 33922052

[CR26] Sato-Dahlman M, Yamamoto M (2018) The development of oncolytic adenovirus therapy in the past and future—for the case of pancreatic cancer. Curr Cancer Drug Targets 18(2):153–161. 10.2174/1568009617666170222123925 28228084 10.2174/1568009617666170222123925 PMC6186423

[CR27] Sung H, Ferlay J, Siegel RL, Laversanne M, Soerjomataram I, Jemal A, Bray F (2021) Global cancer statistics 2020: GLOBOCAN estimates of incidence and mortality worldwide for 36 cancers in 185 countries. CA Cancer J Clin 71(3):209–249. 10.3322/caac.21660 33538338 10.3322/caac.21660

[CR28] Timmer FEF, Geboers B, Nieuwenhuizen S, Dijkstra M, Schouten EAC, Puijk RS, de Vries JJJ, van den Tol MP, Bruynzeel AME, Streppel MM, Wilmink JW, van der Vliet HJ, Meijerink MR, Scheffer HJ, de Gruijl TD (2021) Pancreatic cancer and immunotherapy: a clinical overview. Cancers (Basel). 10.3390/cancers13164138 10.3390/cancers13164138 PMC8393975 34439292

[CR29] Van Cutsem E, Tempero MA, Sigal D, Oh DY, Fazio N, Macarulla T, Hitre E, Hammel P, Hendifar AE, Bates SE, Li CP, Hingorani SR, de la Fouchardiere C, Kasi A, Heinemann V, Maraveyas A, Bahary N, Layos L, Sahai V, Investigators H (2020) Randomized phase III trial of pegvorhyaluronidase alfa with nab-paclitaxel plus gemcitabine for patients with hyaluronan-high metastatic pancreatic adenocarcinoma. J Clin Oncol 38(27):3185–3194. 10.1200/JCO.20.00590 10.1200/JCO.20.00590 PMC7499614 32706635

[CR30] Von Hoff DD, Ervin T, Arena FP, Chiorean EG, Infante J, Moore M, Seay T, Tjulandin SA, Ma WW, Saleh MN, Harris M, Reni M, Dowden S, Laheru D, Bahary N, Ramanathan RK, Tabernero J, Hidalgo M, Goldstein D, Renschler MF (2013) Increased survival in pancreatic cancer with nab-paclitaxel plus gemcitabine. N Engl J Med 369(18):1691–1703. 10.1056/NEJMoa1304369 10.1056/NEJMoa1304369 PMC4631139 24131140

[CR31] Versteijne E, Suker M, Groothuis K, Akkermans-Vogelaar JM, Besselink MG, Bonsing BA, Buijsen J, Busch OR, Creemers GM, van Dam RM, Eskens F, Festen S, de Groot JWB, Groot Koerkamp B, de Hingh IH, Homs MYV, van Hooft JE, Kerver ED, Luelmo SAC, Dutch Pancreatic Cancer G (2020) Preoperative chemoradiotherapy versus immediate surgery for resectable and borderline resectable pancreatic cancer: results of the dutch randomized phase III PREOPANC trial. J Clin Oncol 38(16):1763–1773. 10.1200/JCO.19.02274 10.1200/JCO.19.02274 PMC8265386 32105518

[CR32] Welfare AIOHA (2022) Cancer data in Australia. Australian Institute of Health and Welfare. Retrieved Oct 31, from https://www.aihw.gov.au/reports/cancer/cancer-data-in-australia

[CR33] Yamamoto Y, Goto N, Miura K, Narumi K, Ohnami S, Uchida H, Miura Y, Yamamoto M, Aoki K (2014) Development of a novel efficient method to construct an adenovirus library displaying random peptides on the fiber knob. Mol Pharm 11(3):1069–1074. 10.1021/mp4005854 24380399 10.1021/mp4005854 PMC4110195

[CR34] Zhu Y-H, Zheng J-H, Jia Q-Y, Duan Z-H, Yao H-F, Yang J, Sun Y-W, Jiang S-H, Liu D-J, Huo Y-M (2022) Immunosuppression, immune escape, and immunotherapy in pancreatic cancer: focused on the tumor microenvironment. Cell Oncol. 10.1007/s13402-022-00741-1 10.1007/s13402-022-00741-1 PMC12974690 36367669

