# Supplementary material for: Oncolytic adenoviruses and the treatment of pancreatic cancer: a review of clinical trials
Source: J Cancer Res Clin Oncol. 2023 Apr 8;149(10):8117–29. doi: 10.1007/s00432-023-04735-w (PMC10374677; doi:10.1007/s00432-023-04735-w)
Supplement: Supplementary file 1 — (PDF 184 KB) [file 432_2023_4735_MOESM1_ESM.pdf]

Oncolytic Adenoviruses and The Treatment of Pancreatic Cancer: A Review Of Clinical Trials

Journal of Cancer Research and Clinical Oncology

Isobel P Taylor [1] and J. Alejandro Lopez[2]

[1] School of Medicine and Dentistry, Griffith University, Gold Coast, Australia.

Email: isobel.taylor@griffithuni.edu.au

ORC ID: 0000-0003-1216-7759

[2] School of Environment and Sciences, Griffith University, Nathan, Australia.

Email: a.Lopez@griffith.edu.au

ORC ID: 0000-0001-9370-1516

Supplementary Table 1: Preclinical Literature Of Modified Adenoviruses For Pancreatic Ductal Adenocarcinoma

Research Target	Type of mutation	Mutation	Explanation	Reference
Selectivity	Deletional	E1B 55kDa deletion	E1B 55kDa region contains genes allowing the binding of p53 required for replication in normal cells. Therefore, deletion prevents replication in cells with normal p53 function.	(Bischoff et al., 1996)
		E1A deletion	E1A region contains genes allowing the binding of pRb. Therefore, deletion ensures viral replication only occurs in cells with deficient pRb, such as tumour cells.	(Fueyo et al., 2000; Heise et al., 2000)
		E1A CR2 deletion	E1A CR2 region contains genes responsible for binding and inactivation of pRb thereby releasing E2F for S-phase entry to allow viral replication	(Bhattacharyya et al., 2011; Cherubini et al., 2011; Dai et al., 2017; Kangasniemi et al., 2012; Oberg et al., 2010)

		E1B 15kDa/19kDa deletion	E1B 15kDa/19kDa region contains genes encoding BCL-2 analogue, which resists death receptor and intrinsically induced apoptosis through p53-dependent and independent mechanisms. Improves efficacy of DNA-damaging chemotherapies through convergence on cellular apoptosis pathways.	(Cherubini et al., 2011; Jones et al., 2008; Leitner et al., 2009; Oberg et al., 2010; Pantelidou et al., 2016)
		E1A + E1B deletion	Double deletion causes improved selectivity and efficiency	(Moore et al., 2007; Rocha-Lima & Raez, 2009)
Selectivity and Targeted Therapies	Transcriptional control	Cyclooxgenase-2 promoter	Pancreatic cancer cells have higher levels of these proteins than normal cells, allowing them to selectivity control the gene expression and genome replication of adenoviruses for targeted therapies	(Armstrong, Arrington, et al., 2012; Armstrong, Davydova, et al., 2012; Hoffmann & Wildner, 2006; LaRocca et al., 2015; Ramirez et al., 2008; Yamamoto et al., 2003)
		CDC25B promoter		(Weber et al., 2015)
		Egr1 promoter		(Koujima et al., 2020)
		Urokinase-type plasminogen activator receptor (uPAR) promoter		(Huch et al., 2009; Jose et al., 2013; Maliandi et al., 2015; Mato-Berciano et al., 2017; Sobrevals et al., 2014)
		Human Telomerase reverse transcriptase (hTERT) promoter		(Kanai et al., 2006; Koujima et al., 2020; Nakao et al., 2011; Onimaru et al., 2010; Shan et al., 2013)
		Modified hTERT (mTERT) promoter		(Kim et al., 2003)
		E2F1 promoter		(Hoffmann & Wildner, 2006)
		Hypoxia response element – mTERT – E2F hybrid promoter		(Li et al., 2018)
		Hypoxia responsive promoter		(Bortolanza et al., 2009; Shan et al., 2013)
		Survivin-promoter		(Yamamoto et al., 2014; Yamamoto et al., 2017)

		CXCR4 promoter		(Chu et al., 2012; Kaliberov et al., 2014)
		Carcino-embryonic antigen		(C. Xu et al., 2012; Zhang et al., 2020)
		KRAS		(Lisiansky et al., 2012)
		Midkine promoter		(Hoffmann & Wildner, 2006; Toyoda et al., 2008)
Increased Infectivity	Receptor Modification	Modified receptor from Ad5 to Ad3	Changes binding from CAR, which has a low expression rate in pancreatic cancer, to Desmoglein-2 protein	(Kangasniemi et al., 2012; LaRocca et al., 2015; Ramirez et al., 2008; Wang et al., 2011)
		RGD	Binds to αV-integrins which are upregulated in pancreatic cancer cells, increased adenoviral selectivity for these cells	(Dai et al., 2017; Kangasniemi et al., 2012; LaRocca et al., 2015; Ramirez et al., 2008; Salzwedel et al., 2018; Xu et al., 2017)
		CKS17	Is a peptide targeting the TGF-β receptor which is expressed on pancreatic cancer cells, pancreatic stellate cells and extracellular matrix, promoting viral replication on neoplastic and non-neoplastic cells within PDAC tumours	(Lucas et al., 2015)
		Neurotensin	Is a neuropeptide targeting the neurotensin receptor which is overexpressed in 75% of PDACs and not present on normal pancreatic cells, increasing tumour-selectivity	(Na et al., 2015)
		Expression of A20FMDV2 and ablation of CAR binding	Changes binding from CAR to αvfl6-integrin, which promotes viral propagation and spread, including into pancreatic stellate cells	(Man et al., 2018; Rodriguez et al., 2022)
Targeted therapies	Various Modifications	IFN-γ	Activates macrophages, stimulates the immune system	(Xie et al., 2013)
		IFN-α	Inhibits angiogenesis, stimulates the immune system and sensitises tumour cells to chemo- and radiotherapy	(Armstrong, Davydova, et al., 2012; LaRocca et al., 2015; Salzwedel et al., 2018)
		IL-12	Inhibits angiogenesis and stimulates the immune system through production of IFN- γ in blood mononuclear cells, T-cell differentiation	(Bortolanza et al., 2009; Jung et al., 2020)

	IL-24	Induces T-cell response, activates immune system	(He et al., 2013)
	Adenoviral death protein (ADP)	Promotes the release of progeny virus by accelerating the lysis and death of the cancer cell	(Freytag et al., 2007; Salzwedel et al., 2018)
	Canstatin	Inhibits angiogenesis leading to increased apoptosis	(He et al., 2009)
	Cystosine deaminase	Converts the prodrug 5-fluorocytosine (5-FC) into a toxic metabolite, leading to cell death	(Freytag et al., 2007)
	Decorin	Decreases the activity of transforming growth factor-β thereby decreasing desmoplasia	(Li et al., 2018; Na et al., 2015)
	Endostatin	Decreases endothelial hyperplasia and angiogenesis	(Shan et al., 2013)
	Granulocyte macrophage colony-stimulating factor (GM-CSF)	Immunostimulatory through enhanced antigen presentation, natural killer cell-mediated killing and cytotoxic T-cell response, promoting immune-mediated oncolysis	(Jung et al., 2020)
	Hsp70	Promotes immune response through antigen presentation, T-cell proliferation and dendritic cell maturation	(C. Xu et al., 2012)
	Herpes simplex virus thymidine kinase	Converts the prodrug ganciclovir into a toxic metabolite, leading to cell death	(Freytag et al., 2007; Jose et al., 2013)
	Human tumour necrosis factor-related apoptosis-inducing ligand (TRAIL)	Induces oncolysis by activating the death receptor-mediated apoptotic pathway through binding with death-inducing ligands. Causes G2/M arrest and increased survivin levels.	(Ge et al., 2017; Han et al., 2016; Zhang et al., 2020)
	Human somatostatin receptor gene 2 (hSSTr2)	Increases TRAIL-induced apoptosis	(Zhang et al., 2009)
	Lipocalin-2	Reverses epithelial-mesenchymal transition and inhibits tumour invasion, metastasis and angiogenesis	(B. Xu et al., 2012)
	Oncostatin M (an IL6 cytokine)	Immunostimulatory	(Nistal-Villan et al., 2015)
	P19	RNAi inhibitor that cleaves miRNA leading to increased viral replication	(Doerner et al., 2022)

	Second mitochondria-derived activator of caspase (Smac)	Inhibits the action of inhibitor of apoptosis proteins (IAPs), therefore preventing resistance of tumour cells to TRAIL and increasing apoptosis	(Ge et al., 2017)
	Uracil phosphoribosyltransferase	Increased 5-FC activity and increased radiotherapy sensitivity	(Hasegawa et al., 2013)
	Short hairpin RNA targeting PKM2	Increased apoptosis induction and reduced autophagy through decreased PKM2 activity	(Xu et al., 2017)
	Short hairpin RNA targeting survivin	Downregulates survivin leading to increased gemcitabine and TRAIL-induced cell death	(Han et al., 2016)
	Single chain fragment against the IL6 receptor	Inhibit IL6 production, leading to reduced TGF-fl1 expression and hence reduced desmoplasia and restored immune function. Prevention of IL-6/GM CSF-mediated myeloid suppressor differentiation. Prevention of upregulation of programmed death ligand 1 (PD-L1) and PD-1 on stimulated dendritic cells.	(Eriksson et al., 2019)
	SiRNA GLi1	Increased angiogenesis and reduced tumour volumes by silencing hedgehog signalling	(Guo et al., 2013)
	SiRNA KRAS	Decreases oncogene expression	(Zhang et al., 2006)
	Suppression of tumorigenicity-13 (ST13)	Increases the activity of Hsp70 in tumour cells, leading to increased immunogenicity	(Zhang et al., 2020)
	Trimerized membrane-bound isoleucine zipper (TMZ) human CD40L	Stimulates the immune system in the tumour microenvironment through dendritic cell and macrophage activation	(Eriksson et al., 2019; Eriksson et al., 2017)
	4-1BBL	Enhanced immunologic memory and expands natural killer cells	(Eriksson et al., 2019; Eriksson et al., 2017)
	Heat shock protein	Increases immune response	(C. Xu et al., 2012)
	MicroRNA-143	Decreases KRAS expression, and so acts as a tumour suppressor	(Hu et al., 2012)
	MicroRNA-222 binding sites	Decrease amount of MicroRNA-222 in the cellular environment which sensitises the tumour to the adenovirus	(Raimondi et al., 2021)

	Relaxin	Increases intratumoural spread of adenovirus through increased collagen degradation	(Jung et al., 2020; Jung et al., 2017)
	P53 upregulated apoptotic factor	Results in increased apoptosis	(Wang et al., 2012)
	Plasminogen activator inhibitor-1	Suppresses tumour progression	(Lupu-Meiri et al., 2012)

Supplementary Table 2: Current/future clinical trials

Identifier number	Title	Adenovirus	Other treatment	Route of administration	Phase	Status (as at Dec 2022)
NCT02705196	LOAd703 Oncolytic Virus Therapy for Pancreatic Cancer	**LOAd703** – adenovirus expressing CD40L and 4-1BBL that activate the CD40 and 4-1BB pathways which stimulate the immune system and modulate the tumour stroma microbiology	Gemcitabine + nab-paclitaxel	Intratumoural	I/II	Recruiting

NCT02894944	Clinical Trial Phase I for Theragene in Combination With Chemotherapy for the Locally Advanced Pancreatic Cancer (Theragene)	**Theragene (Ad5-yCD/mutTKSR39rep-ADP) -** adenovirus expressing yeast cytosine deaminase, mutant sr39 HSV thymidine kinase, 11.6 kDa ADP genes	Chemotherapy	Intratumoural	I	Recruitment completed
NCT04739046	An Exploratory Trial to Evaluate Efficacy and Safety for Combination Treatment of Adenovirus Double Suicide Gene Therapy	**Theragene (Ad5-yCD/mutTKSR39rep-ADP) -** adenovirus expressing yeast cytosine deaminase, mutant sr39 HSV thymidine kinase, 11.6 kDa ADP genes	Radiation	Intratumoural	IIa	Recruiting
NCT05076760	Study of MEM-288 Oncolytic Virus in Solid Tumors Including Non-Small Cell Lung Cancer (NSCLC)	**MEM-288 -** conditionally replicative adenovirus encoding transgenes for interferon beta and CD40-ligand	N/A	Intratumoural	I	Recruiting

References

Armstrong, L., Arrington, A., Han, J., Gavrikova, T., Brown, E., Yamamoto, M., Vickers, S. M., & Davydova, J. (2012). Generation of a novel, cyclooxygenase-2-targeted, interferon-expressing, conditionally replicative adenovirus for pancreatic cancer therapy. *Am J Surg*, *204*(5), 741-750. https://doi.org/10.1016/j.amjsurg.2012.02.016

Armstrong, L., Davydova, J., Brown, E., Han, J., Yamamoto, M., & Vickers, S. M. (2012). Delivery of interferon alpha using a novel Cox2-controlled adenovirus for pancreatic cancer therapy. *Surgery*, *152*(1), 114-122. https://doi.org/10.1016/j.surg.2012.02.017

Bhattacharyya, M., Francis, J., Eddouadi, A., Lemoine, N. R., & Hallden, G. (2011). An oncolytic adenovirus defective in pRb-binding (dl922-947) can efficiently eliminate pancreatic cancer cells and tumors in vivo in combination with 5-FU or gemcitabine. *Cancer Gene Ther*, *18*(10), 734-743. https://doi.org/10.1038/cgt.2011.45

Bischoff, J. R., Kirn, D. H., Williams, A., Heise, C., Horn, S., Muna, M., Ng, L., Nye, J. A., Sampson-Johannes, A., Fattaey, A., & McCormick, F. (1996). An adenovirus mutant that replicates selectively in p53-deficient human tumor cells. *Science*, *274*(5286), 373-376. https://doi.org/10.1126/science.274.5286.373

Bortolanza, S., Bunuales, M., Otano, I., Gonzalez-Aseguinolaza, G., Ortiz-de-Solorzano, C., Perez, D., Prieto, J., & Hernandez-Alcoceba, R. (2009). Treatment of pancreatic cancer with an oncolytic adenovirus expressing interleukin-12 in Syrian hamsters. *Mol Ther*, *17*(4), 614-622. https://doi.org/10.1038/mt.2009.9

Cherubini, G., Kallin, C., Mozetic, A., Hammaren-Busch, K., Muller, H., Lemoine, N. R., & Hallden, G. (2011). The oncolytic adenovirus AdDeltaDelta enhances selective cancer cell killing in combination with DNA-damaging drugs in pancreatic cancer models. *Gene Ther*, *18*(12), 1157-1165. https://doi.org/10.1038/gt.2011.141

Chu, Q. D., Sun, G., Pope, M., Luraguiz, N., Curiel, D. T., Kim, R., Li, B. D., & Mathis, J. M. (2012). Virotherapy using a novel chimeric oncolytic adenovirus prolongs survival in a human pancreatic cancer xenograft model. *Surgery*, *152*(3), 441-448. https://doi.org/10.1016/j.surg.2012.05.040

Dai, B., Roife, D., Kang, Y., Gumin, J., Rios Perez, M. V., Li, X., Pratt, M., Brekken, R. A., Fueyo-Margareto, J., Lang, F. F., & Fleming, J. B. (2017). Preclinical Evaluation of Sequential Combination of Oncolytic Adenovirus Delta-24-RGD and Phosphatidylserine-Targeting Antibody in Pancreatic Ductal Adenocarcinoma. *Mol Cancer Ther*, *16*(4), 662-670. https://doi.org/10.1158/1535-7163.MCT-16-0526

Doerner, J., Sallard, E., Zhang, W., Solanki, M., Liu, J., Ehrke-Schulz, E., Zirngibl, H., Lieber, A., & Ehrhardt, A. (2022). Novel Group C Oncolytic Adenoviruses Carrying a miRNA Inhibitor Demonstrate Enhanced Oncolytic Activity In Vitro and In Vivo. *Mol Cancer Ther*, *21*(3), 460-470. https://doi.org/10.1158/1535-7163.MCT-21-0240

Eriksson, E., Milenova, I., Wenthe, J., Moreno, R., Alemany, R., & Loskog, A. (2019). IL-6 Signaling Blockade during CD40-Mediated Immune Activation Favors Antitumor Factors by Reducing TGF-beta, Collagen Type I, and PD-L1/PD-1. *J Immunol*, *202*(3), 787-798. https://doi.org/10.4049/jimmunol.1800717

Eriksson, E., Milenova, I., Wenthe, J., Stahle, M., Leja-Jarblad, J., Ullenhag, G., Dimberg, A., Moreno, R., Alemany, R., & Loskog, A. (2017). Shaping the Tumor Stroma and Sparking Immune Activation by CD40 and 4-1BB Signaling Induced by an Armed Oncolytic Virus. *Clin Cancer Res*, *23*(19), 5846-5857. https://doi.org/10.1158/1078-0432.CCR-17-0285

Freytag, S. O., Barton, K. N., Brown, S. L., Narra, V., Zhang, Y., Tyson, D., Nall, C., Lu, M., Ajlouni, M., Movsas, B., & Kim, J. H. (2007). Replication-competent adenovirus-mediated suicide gene therapy with radiation in a preclinical model of pancreatic cancer. *Mol Ther*, *15*(9), 1600-1606. https://doi.org/10.1038/sj.mt.6300212

Fueyo, J., Gomez-Manzano, C., Alemany, R., Lee, P. S., McDonnell, T. J., Mitlianga, P., Shi, Y. X., Levin, V. A., Yung, W. K., & Kyritsis, A. P. (2000). A mutant oncolytic adenovirus targeting the Rb pathway produces anti-glioma effect in vivo. *Oncogene*, *19*(1), 2-12. https://doi.org/10.1038/sj.onc.1203251

Ge, Y., Lei, W., Ma, Y., Wang, Y., Wei, B., Chen, X., Ru, G., He, X., Mou, X., & Wang, S. (2017). Synergistic antitumor effects of CDK inhibitor SNS032 and an oncolytic adenovirus coexpressing TRAIL and Smac in pancreatic cancer. *Mol Med Rep*, *15*(6), 3521-3528. https://doi.org/10.3892/mmr.2017.6472

Guo, J., Gao, J., Li, Z., Gong, Y., Man, X., Jin, J., & Wu, H. (2013). Adenovirus vector-mediated Gli1 siRNA induces growth inhibition and apoptosis in human pancreatic cancer with Smo-dependent or Smo-independent Hh pathway activation in vitro and in vivo. *Cancer Lett*, *339*(2), 185-194. https://doi.org/10.1016/j.canlet.2013.06.010

Han, Z., Lee, S., Je, S., Eom, C. Y., Choi, H. J., Song, J. J., & Kim, J. H. (2016). Survivin silencing and TRAIL expression using oncolytic adenovirus increase anti-tumorigenic activity in gemcitabine-resistant pancreatic cancer cells. *Apoptosis*, *21*(3), 351-364. https://doi.org/10.1007/s10495-015-1208-z

Hasegawa, N., Abei, M., Yokoyama, K. K., Fukuda, K., Seo, E., Kawashima, R., Nakano, Y., Yamada, T., Nakade, K., Hamada, H., Obata, Y., & Hyodo, I. (2013). Cyclophosphamide enhances antitumor efficacy of oncolytic adenovirus expressing uracil phosphoribosyltransferase (UPRT) in immunocompetent Syrian hamsters. *Int J Cancer*, *133*(6), 1479-1488. https://doi.org/10.1002/ijc.28132

He, B., Huang, X., Liu, X., & Xu, B. (2013). Cancer targeting gene-viro-therapy for pancreatic cancer using oncolytic adenovirus ZD55-IL-24 in immune-competent mice. *Mol Biol Rep*, *40*(9), 5397-5405. https://doi.org/10.1007/s11033-013-2638-8

He, X. P., Su, C. Q., Wang, X. H., Pan, X., Tu, Z. X., Gong, Y. F., Gao, J., Liao, Z., Jin, J., Wu, H. Y., Man, X. H., & Li, Z. S. (2009). E1B-55kD-deleted oncolytic adenovirus armed with canstatin gene yields an enhanced anti-tumor efficacy on pancreatic cancer. *Cancer Lett*, *285*(1), 89-98. https://doi.org/10.1016/j.canlet.2009.05.006

Heise, C., Hermiston, T., Johnson, L., Brooks, G., Sampson-Johannes, A., Williams, A., Hawkins, L., & Kirn, D. (2000). An adenovirus E1A mutant that demonstrates potent and selective systemic anti-tumoral efficacy. *Nat Med*, *6*(10), 1134-1139. https://doi.org/10.1038/80474

Hoffmann, D., & Wildner, O. (2006). Restriction of adenoviral replication to the transcriptional intersection of two different promoters for colorectal and pancreatic cancer treatment. *Mol Cancer Ther*, *5*(2), 374-381. https://doi.org/10.1158/1535-7163.MCT-05-0374

Hu, Y., Ou, Y., Wu, K., Chen, Y., & Sun, W. (2012). miR-143 inhibits the metastasis of pancreatic cancer and an associated signaling pathway. *Tumour Biol*, *33*(6), 1863-1870. https://doi.org/10.1007/s13277-012-0446-8

Huch, M., Gros, A., Jose, A., Gonzalez, J. R., Alemany, R., & Fillat, C. (2009). Urokinase-type plasminogen activator receptor transcriptionally controlled adenoviruses eradicate pancreatic tumors and liver metastasis in mouse models. *Neoplasia*, *11*(6), 518-528, 514 p following 528. https://doi.org/10.1593/neo.81674

Jones, S., Zhang, X., Parsons, D. W., Lin, J. C., Leary, R. J., Angenendt, P., Mankoo, P., Carter, H., Kamiyama, H., Jimeno, A., Hong, S. M., Fu, B., Lin, M. T., Calhoun, E. S., Kamiyama, M., Walter, K., Nikolskaya, T., Nikolsky, Y., Hartigan, J., . . . Kinzler, K. W. (2008). Core signaling pathways in human pancreatic cancers revealed by global genomic analyses. *Science*, *321*(5897), 1801-1806. https://doi.org/10.1126/science.1164368

Jose, A., Sobrevals, L., Miguel Camacho-Sanchez, J., Huch, M., Andreu, N., Ayuso, E., Navarro, P., Alemany, R., & Fillat, C. (2013). Intraductal delivery of adenoviruses targets pancreatic tumors in transgenic Ela-myc mice and orthotopic xenografts. *Oncotarget*, *4*(1), 94-105. https://doi.org/10.18632/oncotarget.795

Jung, B. K., Ko, H. Y., Kang, H., Hong, J., Ahn, H. M., Na, Y., Kim, H., Kim, J. S., & Yun, C. O. (2020). Relaxin-expressing oncolytic adenovirus induces remodeling of physical and immunological aspects of cold tumor to potentiate PD-1 blockade. *J Immunother Cancer*, *8*(2). https://doi.org/10.1136/jitc-2020-000763

Jung, K. H., Choi, I. K., Lee, H. S., Yan, H. H., Son, M. K., Ahn, H. M., Hong, J., Yun, C. O., & Hong, S. S. (2017). Oncolytic adenovirus expressing relaxin (YDC002) enhances therapeutic efficacy of gemcitabine against pancreatic cancer. *Cancer Lett*, *396*, 155-166. https://doi.org/10.1016/j.canlet.2017.03.009

Kaliberov, S. A., Kaliberova, L. N., Buchsbaum, D. J., & Curiel, D. T. (2014). Experimental virotherapy of chemoresistant pancreatic carcinoma using infectivity-enhanced fiber-mosaic oncolytic adenovirus. *Cancer Gene Ther*, *21*(7), 264-274. https://doi.org/10.1038/cgt.2014.26

Kanai, R., Tomita, H., Shinoda, A., Takahashi, M., Goldman, S., Okano, H., Kawase, T., & Yazaki, T. (2006). Enhanced therapeutic efficacy of G207 for the treatment of glioma through Musashi1 promoter retargeting of gamma34.5-mediated virulence. *Gene Ther*, *13*(2), 106-116. https://doi.org/10.1038/sj.gt.3302636

Kangasniemi, L., Parviainen, S., Pisto, T., Koskinen, M., Jokinen, M., Kiviluoto, T., Cerullo, V., Jalonen, H., Koski, A., Kangasniemi, A., Kanerva, A., Pesonen, S., & Hemminki, A. (2012). Effects of capsid-modified oncolytic adenoviruses and their combinations with gemcitabine or silica gel on pancreatic cancer. *Int J Cancer*, *131*(1), 253-263. https://doi.org/10.1002/ijc.26370

Kim, E., Kim, J. H., Shin, H. Y., Lee, H., Yang, J. M., Kim, J., Sohn, J. H., Kim, H., & Yun, C. O. (2003). Ad-mTERT-delta19, a conditional replication-competent adenovirus driven by the human telomerase promoter, selectively replicates in and elicits cytopathic effect in a cancer cell-specific manner. *Hum Gene Ther*, *14*(15), 1415-1428. https://doi.org/10.1089/104303403769211637

Koujima, T., Tazawa, H., Ieda, T., Araki, H., Fushimi, T., Shoji, R., Kuroda, S., Kikuchi, S., Yoshida, R., Umeda, Y., Teraishi, F., Urata, Y., Mizuguchi, H., & Fujiwara, T. (2020). Oncolytic Virus-Mediated Targeting of the ERK Signaling Pathway Inhibits Invasive Propensity in Human Pancreatic Cancer. *Mol Ther Oncolytics*, *17*, 107-117. https://doi.org/10.1016/j.omto.2020.03.016

LaRocca, C. J., Han, J., Gavrikova, T., Armstrong, L., Oliveira, A. R., Shanley, R., Vickers, S. M., Yamamoto, M., & Davydova, J. (2015). Oncolytic adenovirus expressing interferon alpha in a syngeneic Syrian hamster model for the treatment of pancreatic cancer. *Surgery*, *157*(5), 888-898. https://doi.org/10.1016/j.surg.2015.01.006

Leitner, S., Sweeney, K., Oberg, D., Davies, D., Miranda, E., Lemoine, N. R., & Hallden, G. (2009). Oncolytic adenoviral mutants with E1B19K gene deletions enhance gemcitabine-induced apoptosis in pancreatic carcinoma cells and anti-tumor efficacy in vivo. *Clin Cancer Res*, *15*(5), 1730-1740. https://doi.org/10.1158/1078-0432.CCR-08-2008

Li, Y., Hong, J., Oh, J. E., Yoon, A. R., & Yun, C. O. (2018). Potent antitumor effect of tumor microenvironment-targeted oncolytic adenovirus against desmoplastic pancreatic cancer. *Int J Cancer*, *142*(2), 392-413. https://doi.org/10.1002/ijc.31060

Lisiansky, V., Naumov, I., Shapira, S., Kazanov, D., Starr, A., Arber, N., & Kraus, S. (2012). Gene therapy of pancreatic cancer targeting the K-Ras oncogene. *Cancer Gene Ther*, *19*(12), 862-869. https://doi.org/10.1038/cgt.2012.73

Lucas, T., Benihoud, K., Vigant, F., Schmidt, C. Q., Wortmann, A., Bachem, M. G., Simmet, T., & Kochanek, S. (2015). Hexon modification to improve the activity of oncolytic adenovirus vectors against neoplastic and stromal cells in pancreatic cancer. *PLoS One*, *10*(2), e0117254. https://doi.org/10.1371/journal.pone.0117254

Lupu-Meiri, M., Geras-Raaka, E., Lupu, R., Shapira, H., Sandbank, J., Segal, L., Gershengorn, M. C., & Oron, Y. (2012). Knock-down of plasminogen-activator inhibitor-1 enhances expression of E-cadherin and promotes epithelial differentiation of human pancreatic adenocarcinoma cells. *J Cell Physiol*, *227*(11), 3621-3628. https://doi.org/10.1002/jcp.24068

Maliandi, M. V., Mato-Berciano, A., Sobrevals, L., Roue, G., Jose, A., & Fillat, C. (2015). AduPARE1A and gemcitabine combined treatment trigger synergistic antitumor effects in pancreatic cancer through NF-kappaB mediated uPAR activation. *Mol Cancer*, *14*, 146. https://doi.org/10.1186/s12943-015-0413-2

Man, Y. K. S., Davies, J. A., Coughlan, L., Pantelidou, C., Blazquez-Moreno, A., Marshall, J. F., Parker, A. L., & Hallden, G. (2018). The Novel Oncolytic Adenoviral Mutant Ad5-3Delta-A20T Retargeted to alphavbeta6 Integrins Efficiently Eliminates Pancreatic Cancer Cells. *Mol Cancer Ther*, *17*(2), 575-587. https://doi.org/10.1158/1535-7163.MCT-17-0671

Mato-Berciano, A., Raimondi, G., Maliandi, M. V., Alemany, R., Montoliu, L., & Fillat, C. (2017). A NOTCH-sensitive uPAR-regulated oncolytic adenovirus effectively suppresses pancreatic tumor growth and triggers synergistic anticancer effects with gemcitabine and nab-paclitaxel. *Oncotarget*, *8*(14), 22700-22715. https://doi.org/10.18632/oncotarget.15169

Moore, M. J., Goldstein, D., Hamm, J., Figer, A., Hecht, J. R., Gallinger, S., Au, H. J., Murawa, P., Walde, D., Wolff, R. A., Campos, D., Lim, R., Ding, K., Clark, G., Voskoglou-Nomikos, T., Ptasynski, M., Parulekar, W., & National Cancer Institute of Canada Clinical Trials, G. (2007). Erlotinib plus gemcitabine compared with gemcitabine alone in patients with advanced pancreatic cancer: a phase III trial of the National Cancer Institute of Canada Clinical Trials Group. *J Clin Oncol*, *25*(15), 1960-1966. https://doi.org/10.1200/JCO.2006.07.9525

Na, Y., Choi, J. W., Kasala, D., Hong, J., Oh, E., Li, Y., Jung, S. J., Kim, S. W., & Yun, C. O. (2015). Potent antitumor effect of neurotensin receptor-targeted oncolytic adenovirus co-expressing decorin and Wnt antagonist in an orthotopic pancreatic tumor model. *J Control Release*, *220*(Pt B), 766-782. https://doi.org/10.1016/j.jconrel.2015.10.015

Nakao, A., Kasuya, H., Sahin, T. T., Nomura, N., Kanzaki, A., Misawa, M., Shirota, T., Yamada, S., Fujii, T., Sugimoto, H., Shikano, T., Nomoto, S., Takeda, S., Kodera, Y., & Nishiyama, Y. (2011). A phase I dose-escalation clinical trial of intraoperative direct intratumoral injection of HF10 oncolytic virus in non-resectable patients with advanced pancreatic cancer. *Cancer Gene Ther*, *18*(3), 167-175. https://doi.org/10.1038/cgt.2010.65

Nistal-Villan, E., Bunuales, M., Poutou, J., Gonzalez-Aparicio, M., Bravo-Perez, C., Quetglas, J. I., Carte, B., Gonzalez-Aseguinolaza, G., Prieto, J., Larrea, E., & Hernandez-Alcoceba, R. (2015). Enhanced therapeutic effect using sequential administration of antigenically distinct oncolytic viruses expressing oncostatin M in a Syrian hamster orthotopic pancreatic cancer model. *Mol Cancer*, *14*, 210. https://doi.org/10.1186/s12943-015-0479-x

Oberg, D., Yanover, E., Adam, V., Sweeney, K., Costas, C., Lemoine, N. R., & Hallden, G. (2010). Improved potency and selectivity of an oncolytic E1ACR2 and E1B19K deleted adenoviral mutant in prostate and pancreatic cancers. *Clin Cancer Res*, *16*(2), 541-553. https://doi.org/10.1158/1078-0432.CCR-09-1960

Onimaru, M., Ohuchida, K., Nagai, E., Mizumoto, K., Egami, T., Cui, L., Sato, N., Uchino, J., Takayama, K., Hashizume, M., & Tanaka, M. (2010). Combination with low-dose gemcitabine and hTERT-promoter-dependent conditionally replicative adenovirus enhances cytotoxicity through their crosstalk mechanisms in pancreatic cancer. *Cancer Lett*, *294*(2), 178-186. https://doi.org/10.1016/j.canlet.2010.01.034

Pantelidou, C., Cherubini, G., Lemoine, N. R., & Hallden, G. (2016). The E1B19K-deleted oncolytic adenovirus mutant AdDelta19K sensitizes pancreatic cancer cells to drug-induced DNA-damage by down-regulating Claspin and Mre11. *Oncotarget*, *7*(13), 15703-15724. https://doi.org/10.18632/oncotarget.7310

Raimondi, G., Gea-Sorlí, S., Otero-Mateo, M., & Fillat, C. (2021). Inhibition of miR-222 by Oncolytic Adenovirus-Encoded miRNA Sponges Promotes Viral Oncolysis and Elicits Antitumor Effects in Pancreatic Cancer Models. *Cancers*, *13*(13), 3233. https://doi.org/10.3390/cancers13133233

Ramirez, P. J., Vickers, S. M., Ono, H. A., Davydova, J., Takayama, K., Thompson, T. C., Curiel, D. T., Bland, K. I., & Yamamoto, M. (2008). Optimization of conditionally replicative adenovirus for pancreatic cancer and its evaluation in an orthotopic murine xenograft model. *Am J Surg*, *195*(4), 481-490. https://doi.org/10.1016/j.amjsurg.2007.04.014

Rocha-Lima, C. M., & Raez, L. E. (2009). Erlotinib (tarceva) for the treatment of non-small-cell lung cancer and pancreatic cancer. *P T*, *34*(10), 554-564. https://www.ncbi.nlm.nih.gov/pubmed/20140116

Rodriguez, M., Rodriguez, I. G., Nattress, C., Qureshi, A., & Hallden, G. (2022). HDAC Inhibitors Enhance Efficacy of the Oncolytic Adenoviruses Ad and Ad-3-A20T in Pancreatic and Triple-Negative Breast Cancer Models. *Viruses*, *14*(5). https://doi.org/10.3390/v14051006

Salzwedel, A. O., Han, J., LaRocca, C. J., Shanley, R., Yamamoto, M., & Davydova, J. (2018). Combination of interferon-expressing oncolytic adenovirus with chemotherapy and radiation is highly synergistic in hamster model of pancreatic cancer. *Oncotarget*, *9*(26), 18041-18052. https://doi.org/10.18632/oncotarget.24710

Shan, Y. F., Fang, Y. F., Wang, X. Q., Jin, R., Zhang, Q. Y., & Andersson, R. (2013). Experimental studies on treatment of pancreatic cancer with double-regulated duplicative adenovirus AdTPHre-hEndo carrying human endostatin gene. *Pancreatology*, *13*(4), 393-400. https://doi.org/10.1016/j.pan.2013.05.012

Sobrevals, L., Mato-Berciano, A., Urtasun, N., Mazo, A., & Fillat, C. (2014). uPAR-controlled oncolytic adenoviruses eliminate cancer stem cells in human pancreatic tumors. *Stem Cell Res*, *12*(1), 1-10. https://doi.org/10.1016/j.scr.2013.09.008

Toyoda, E., Doi, R., Kami, K., Mori, T., Ito, D., Koizumi, M., Kida, A., Nagai, K., Ito, T., Masui, T., Wada, M., Tagawa, M., & Uemoto, S. (2008). Midkine promoter-based conditionally replicative adenovirus therapy for midkine-expressing human pancreatic cancer. *J Exp Clin Cancer Res*, *27*, 30. https://doi.org/10.1186/1756-9966-27-30

Wang, H., Li, Z. Y., Liu, Y., Persson, J., Beyer, I., Moller, T., Koyuncu, D., Drescher, M. R., Strauss, R., Zhang, X. B., Wahl, J. K., 3rd, Urban, N., Drescher, C., Hemminki, A., Fender, P., & Lieber, A. (2011). Desmoglein 2 is a receptor for adenovirus serotypes 3, 7, 11 and 14. *Nat Med*, *17*(1), 96-104. https://doi.org/10.1038/nm.2270

Wang, H., Pei, W., Luan, Q., Ma, F., Zhou, S., Zhao, Z., Meng, X., Zhang, X., Liang, X., Chen, Y., Zhan, Q., Lin, C., Qian, H., & Zhao, P. (2012). A feasibility study on gene therapy of pancreatic carcinoma with Ad-PUMA. *Cancer Biol Ther*, *13*(9), 712-719. https://doi.org/10.4161/cbt.20552

Weber, H. L., Gidekel, M., Werbajh, S., Salvatierra, E., Rotondaro, C., Sganga, L., Haab, G. A., Curiel, D. T., Cafferata, E. G., & Podhajcer, O. L. (2015). A Novel CDC25B Promoter-Based Oncolytic Adenovirus Inhibited Growth of Orthotopic Human Pancreatic Tumors in Different Preclinical Models. *Clin Cancer Res*, *21*(7), 1665-1674. https://doi.org/10.1158/1078-0432.CCR-14-2316

Xie, F. J., Zhao, P., Zhang, Y. P., Liu, F. Y., Nie, X. L., Zhu, Y. H., Yu, X. M., Zheng, Q. Q., Mao, W. M., Lu, H. Y., Wei, H., & Huang, W. (2013). Adenovirus-mediated interferon-gamma gene therapy induced human pancreatic carcinoma Capan-2 cell apoptosis in vitro and in vivo. *Anat Rec (Hoboken)*, *296*(4), 604-610. https://doi.org/10.1002/ar.22661

Xu, B., Zheng, W. Y., Jin, D. Y., Wang, D. S., Liu, X. Y., & Qin, X. Y. (2012). Treatment of pancreatic cancer using an oncolytic virus harboring the lipocalin-2 gene. *Cancer*, *118*(21), 5217-5226. https://doi.org/10.1002/cncr.27535

Xu, C., Sun, Y., Wang, Y., Yan, Y., Shi, Z., Chen, L., Lin, H., Lu, S., Zhu, M., Su, C., & Li, Z. (2012). CEA promoter-regulated oncolytic adenovirus-mediated Hsp70 expression in immune gene therapy for pancreatic cancer. *Cancer Lett*, *319*(2), 154-163. https://doi.org/10.1016/j.canlet.2012.01.009

Xu, Y., Chu, L., Yuan, S., Yang, Y., Yang, Y., Xu, B., Zhang, K., Liu, X. Y., Wang, R., Fang, L., Chen, Z., & Liang, Z. (2017). RGD-modified oncolytic adenovirus-harboring shPKM2 exhibits a potent cytotoxic effect in pancreatic cancer via autophagy inhibition and apoptosis promotion. *Cell Death Dis*, *8*(6), e2835. https://doi.org/10.1038/cddis.2017.230

Yamamoto, M., Davydova, J., Wang, M., Siegal, G. P., Krasnykh, V., Vickers, S. M., & Curiel, D. T. (2003). Infectivity enhanced, cyclooxygenase-2 promoter-based conditionally replicative adenovirus for pancreatic cancer. *Gastroenterology*, *125*(4), 1203-1218. https://doi.org/10.1016/s0016-5085(03)01196-x

Yamamoto, Y., Hiraoka, N., Goto, N., Rin, Y., Miura, K., Narumi, K., Uchida, H., Tagawa, M., & Aoki, K. (2014). A targeting ligand enhances infectivity and cytotoxicity of an oncolytic adenovirus in human pancreatic cancer tissues. *J Control Release*, *192*, 284-293. https://doi.org/10.1016/j.jconrel.2014.07.053

Yamamoto, Y., Nagasato, M., Rin, Y., Henmi, M., Ino, Y., Yachida, S., Ohki, R., Hiraoka, N., Tagawa, M., & Aoki, K. (2017). Strong antitumor efficacy of a pancreatic tumor-targeting oncolytic adenovirus for neuroendocrine tumors. *Cancer Med*, *6*(10), 2385-2397. https://doi.org/10.1002/cam4.1185

Zhang, Y., Ye, M., Huang, F., Wang, S., Wang, H., Mou, X., & Wang, Y. (2020). Oncolytic Adenovirus Expressing ST13 Increases Antitumor Effect of Tumor Necrosis Factor-Related Apoptosis-Inducing Ligand Against Pancreatic Ductal Adenocarcinoma. *Hum Gene Ther*, *31*(15-16), 891-903. https://doi.org/10.1089/hum.2020.024

Zhang, Y. A., Nemunaitis, J., Samuel, S. K., Chen, P., Shen, Y., & Tong, A. W. (2006). Antitumor activity of an oncolytic adenovirus-delivered oncogene small interfering RNA. *Cancer Res*, *66*(19), 9736-9743. https://doi.org/10.1158/0008-5472.CAN-06-1617

Zhang, Z., Huang, Y., Newman, K., Gu, J., Zhang, X., Wu, H., Zhao, M., Xianyu, Z., & Liu, X. (2009). Reexpression of human somatostatin receptor gene 2 gene mediated by oncolytic adenovirus increases antitumor activity of tumor necrosis factor-related apoptosis-inducing ligand against pancreatic cancer. *Clin Cancer Res*, *15*(16), 5154-5160. https://doi.org/10.1158/1078-0432.CCR-09-0025
